# Mitigating polarization in flat-sheet membrane distillation through CFD-driven spacer design

**DOI:** 10.1038/s41598-026-51007-x

**Published:** 2026-05-07

**Authors:** Sara Karimi, Matteo Morciano, Carlos Plana Turmo, Matteo Maria Piredda, Pietro Asinari, Oliver Gloth, Matteo Fasano

**Affiliations:** 1 Department of Energy, Politecnico di Torino, Corso Duca degli Abruzzi 24, 10129 Torino, Italy; 2 Clean Water Center, Politecnico di Torino, Corso Duca degli Abruzzi 24, 10129 Torino, Italy; 3 Istituto Nazionale di Ricerca Metrologica, Strada delle Cacce 91, 10135 Torino, Italy; 4 enGits GmbH, Langenbachstrasse 3, 79674 Todtnau, Germany

**Keywords:** Energy science and technology, Engineering, Mathematics and computing

## Abstract

Membrane distillation (MD) is a thermally driven separation process that has attracted attention for its ability to couple with low-grade or renewable heat sources, making it well suited for sustainable water desalination amid rising freshwater demand and climate change pressures. A key open issue remains the optimization of fluid-dynamics within flat-sheet MD modules to minimize flow maldistribution and maximize both heat and mass transfer, and thus module performance. In this work, we numerically investigate and optimize spacer geometries – components that play a critical role in governing these transport processes. Here, using a validated computational fluid dynamics (CFD) approach, we simulate the hydrodynamics and thermal behavior of the direct contact membrane distillation (DCMD) process. Our model is first validated against published experimental data, ensuring that predicted temperature and velocity fields match observed performance metrics. We then conduct an extensive parametric study across a range of novel spacer designs – varying filament shape, orientation, and spacing – to assess their influence on flow uniformity, temperature and concentration polarization, and overall thermal efficiency. Compared to the best performing spacer configuration reported in the literature, our twisted and elliptical spacer geometries achieve up to a 5.4% reduction in temperature polarization coefficient, indicating a measurable enhancement in heat and mass transfer efficiency. These findings provide a clear roadmap for future experimental implementation of innovative spacers in MD modules, with the goal of significantly improving desalination performance and reducing energy consumption.

## Introduction

The scarcity of safe drinking water is one of the most pressing global challenges of the 21st century. According to the United Nations, by 2050 nearly half of the world’s population is expected to experience some degree of water stress or scarcity. In this scenario, the growing demand for potable water, together with the looming effects of climate change and the urgent need to exploit renewable resources, highlights the importance of developing innovative, sustainable solutions for water treatment and desalination^1^.

Among the available technologies, membrane distillation (MD) has emerged as a particularly promising thermally driven separation process. Its advantages include operation at relatively low temperatures, nearly complete salt rejection, and compatibility with low-grade or renewable heat sources^2,3^. In its most widely studied configuration, direct contact membrane distillation (DCMD), hot saline feed water and cold permeate streams are separated by a hydrophobic membrane, and mass transfer is driven by the vapor pressure gradient across the membrane.

Despite these advantages, the large-scale deployment of MD remains hindered by key limitations, most notably temperature and concentration polarization^4–6^. These phenomena reduce the effective transmembrane driving force, lowering permeate flux and overall process efficiency^7–9^. Moreover, polarization phenomena not only reduce permeate flux but can also promote fouling and scaling at the membrane interface. Regions with high concentration polarization and local temperature gradients are particularly prone to solute accumulation, which may accelerate deposit formation^10^. To minimize these effects, feed and permeate channels are often equipped with spacers – typically net-type polymeric structures – fabricated for example from polypropylene^11^ or ABS (Acrylonitrile Butadiene Styrene)^8^. In addition to maintaining channel spacing and providing mechanical support to the membrane^12^, spacers enhance local mixing, thereby limiting polarization effects^13^. However, these benefits frequently come at the cost of higher pressure drops and the creation of hydrodynamic dead zones, strongly dependent on spacer design^9^.

Feed channel spacer design has been extensively investigated in pressure driven membrane processes, e.g. reverse osmosis (RO), nanofiltration (NF) or ultrafiltration (UF), where spacers play a central role in mitigating concentration polarization and fouling while inevitably increasing pressure drop. Foundational experimental and modeling studies by Schock and Miquel^14^ and by da Costa et al.^15^ demonstrated that spacer-induced mixing enhances mass transfer but introduces a fundamental trade-off between polarization reduction and hydraulic losses. Later, this trade-off has been actively revisited using advanced computational fluid dynamics and additive manufacturing. Lin et al.^16^ showed that more uniform cylindrical filaments reduce dead zones and fouling propensity, while Park et al.^17^ demonstrated that 3D-printed honeycomb spacers can reduce foulant layer thickness by approximately 30% compared to conventional meshes. More recently, Qamar et al.^18^ and Chong et al.^19^ reported innovative pillar-based and twisted spacers that simultaneously increase mass transfer coefficients (up to 50% higher Sherwood number) and reduce pressure drop relative to commercial designs. Although MD differs from RO in being thermally rather than pressure driven, these studies establish general design principles, namely boundary-layer disruption, flow redistribution, and dead-zone minimization, that are directly transferable to MD spacer optimization, where enhanced mixing primarily targets temperature and concentration polarization with comparatively minor pumping energy penalties.

Recent advances in additive manufacturing have further expanded the design possibilities for membrane spacers. Traditional polymeric spacers are typically produced via extrusion or net-casting techniques; however, these methods inherently limit geometric flexibility and surface precision. In contrast, 3D printing enables rapid prototyping of complex structures with tailored hydrodynamic characteristics, allowing researchers to systematically tune filament topology, surface morphology, and porosity to improve performance and limit fouling. Early work demonstrated the potential of additively manufactured spacers to enhance mass transfer compared to commercial mesh designs^20^. More recently, triply periodic minimal surface (TPMS) geometries and other architected structures produced via 3D printing have shown significant improvements in heat and mass transfer, polarization control, and fouling resistance^21,22^. These advances highlight additive manufacturing as a key enabler for next-generation spacer architectures, capable of synergistically boosting transport efficiency while supporting membrane integrity and process stability.

Recent spacer innovations in membrane distillation have leveraged helical and spiral configurations to enhance performance by intensifying mixing and mitigating polarization. Helical designs, such as the “strake” insert studied by Al-Muallem et al.^23^, generate swirling flow that disrupts thermal and concentration boundary layers in vacuum tube MD, resulting in a 44% increase in permeate flux – though at the cost of increased pressure drop due to induced turbulence. Spiral configurations, such as the coiled spacer developed by Ibrahim et al.^24^, offer a gentler flow modulation that minimizes pressure drop and pumping energy, albeit with less turbulence-induced enhancement. These spacer architectures, often enabled by additive manufacturing, demonstrate the critical role of geometry in optimizing MD module performance.

Consequently, recent research has increasingly focused on spacer optimization to balance improved heat and mass transfer with minimal hydraulic losses. Both experimental and numerical investigations have shown that parameters such as spacer orientation, filament diameter, and flow attack angle critically affect shear stress distribution, local velocity fields, and ultimately the severity of temperature polarization^3,9^. Complex configurations, including cross-diagonal arrangements with tuned hydrodynamic angles, carbon-fiber based structures, and multi-layer spacers, have demonstrated significant disruption of thermal boundary layers, resulting in flux enhancements of up to 45% in some cases^3^.

Computational fluid dynamics (CFD) has established itself as a powerful tool for exploring and optimizing such designs. CFD provides detailed insight into coupled flow, heat, and mass transfer processes that are difficult to capture experimentally, particularly in narrow channels structured with spacers^25–27^. Existing studies have developed both two- and three-dimensional CFD models of MD systems, often incorporating membrane permeation, simultaneous heat and mass transfer, turbulence modeling, and even coupling with solar-assisted operation^2,9^.

Nevertheless, the design space of possible spacer geometries remains vast and largely unexplored. While prior work has provided valuable understanding of conventional layouts, there is still a need for systematic studies of novel geometries capable of redistributing flow more effectively and reducing polarization effects under realistic, energy-constrained operating conditions. The present work addresses this gap by developing a CFD framework to evaluate the performance of various spacer configurations. A comparative analysis is conducted to identify geometries that provide the most favorable balance of hydrodynamic and thermal performance.

Beyond its methodological scope, the study highlights spacer geometries that mitigate polarization with limited energy penalties, thereby facilitating the integration of MD with renewable and low-grade heat sources. These advances help bridge the gap between laboratory investigations and practical desalination systems, contributing to water production strategies that are both efficient and sustainable.

## Theoretical background

### Polarization effects

**Figure 1:**
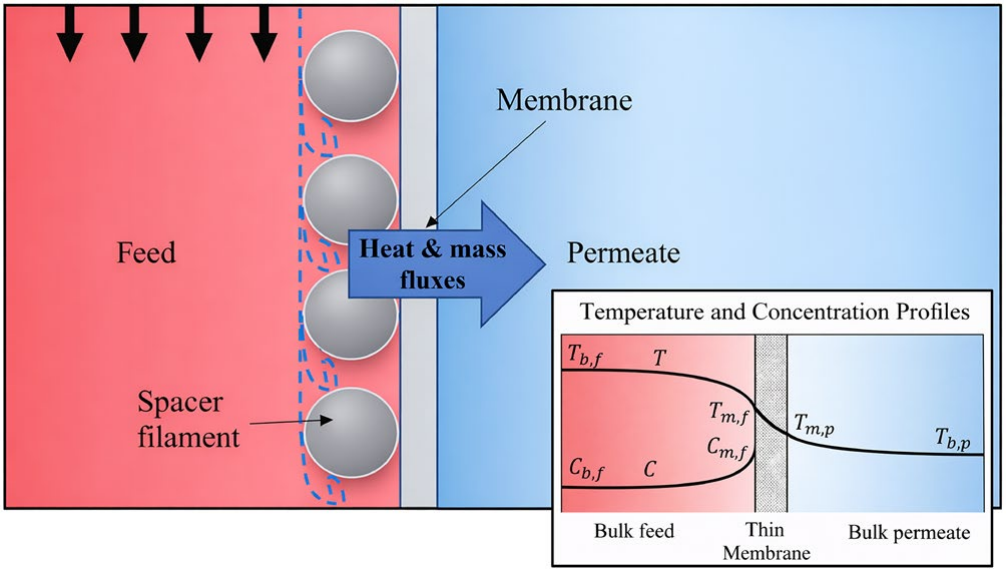
Schematic representation of coupled heat and mass transfer across a spacer-filled membrane module. The feed stream (left) and permeate stream (right) are separated by a porous membrane. Spacer filaments enhance hydrodynamic mixing and alleviate temperature and concentration polarization. The arrow indicates the direction of the transmembrane heat and mass fluxes. The dashed blue line depicts vortical structures induced by the spacer, illustrating secondary flows that disrupt boundary layers and promote local mixing near the membrane surface. The inset shows the corresponding temperature (T) and concentration (C) polarization profiles, highlighting gradients between bulk phases and membrane interfaces.

Figure 1 illustrates the main heat and mass transfer phenomena in a DCMD system. The hot saline feed flows in direct contact with the hydrophobic membrane, while the cold permeate flows on the opposite side. The driving force is the vapor pressure difference across the membrane, which depends on both thermal and concentration gradients. Two polarization phenomena reduce the effective driving force: temperature polarization and concentration polarization.

Temperature polarization occurs because the feed solution adjacent to the membrane cools down due to evaporation, while the permeate-side surface warms up due to condensation. As a result, the actual membrane surface temperatures ($T_{m,f}$ on the feed side and $T_{m,p}$ on the permeate side) deviate from the bulk temperatures ($T_{b,f}$ and $T_{b,p}$). This reduces the effective temperature gradient across the membrane and, consequently, the distillation efficiency. The temperature polarization coefficient (TPC) quantifies this effect^28^:

1 $$\begin{aligned} TPC = \frac{T_{m,f}-T_{m,p}}{T_{b,f}-T_{b,p}}. \end{aligned}$$

A low *TPC* indicates poor heat transfer efficiency and reduced vapor driving force. Thus, improving fluid mixing, e.g. with spacers, is critical to increase *TPC* and enhance process performance.

Concentration polarization arises on the feed side, where non-volatile solutes accumulate near the membrane surface. The solute concentration at the membrane ($C_{m,f}$) exceeds the bulk feed concentration ($C_{b,f}$), reducing the effective vapor pressure gradient and lowering flux. The concentration polarization coefficient (CPC) is defined as^29^:

2 $$\begin{aligned} CPC = \frac{C_{m,f}}{C_{b,f}}. \end{aligned}$$

Values of $CPC > 1$ indicate concentration polarization, with stronger accumulation leading to greater performance losses. Mitigating concentration polarization, again through mixing promoted by spacers, improves mass transfer efficiency.

Both polarization phenomena act to reduce the effective driving force of the DCMD process. Spacer geometries are designed not only to maintain channel spacing and mechanical stability, but also to enhance mixing and thereby limit polarization effects. This makes spacer design a central factor in optimizing membrane distillation modules.

### Distillation performance

Alongside polarization phenomena, the normalized permeate flux is a key metric to evaluate spacer performance^30^. It is defined as

3 $$\begin{aligned} J' = \frac{J}{J_0} = \frac{\int _A B \left( T_{m,f}-T_{b,p}\right) dA}{\int _A B \left( T_{b,f}-T_{b,p}\right) dA}, \end{aligned}$$

where *J* is the actual permeate flux, $J_0$ is the maximum attainable flux, *A* is the membrane surface, and *B* is the mass transfer coefficient.

In general, the distillate flow depends on the intrinsic properties of the membrane as well as on the transmembrane driving force, i.e. the vapor pressure difference across the membrane. The local permeate flux can therefore be expressed as

4 $$\begin{aligned} J = \int _A C_m \left( p_f - p_p\right) dA, \end{aligned}$$

where $C_m$ is the membrane permeability coefficient and $p_f$ and $p_p$ are the vapor pressures on the feed and permeate sides, respectively. Because temperature is more readily measured than vapor pressure, Eq. 4 is often rewritten as

5 $$\begin{aligned} J = \int _A B \left( T_{m,f} - T_{m,p}\right) dA. \end{aligned}$$

The mass transfer coefficient *B* is related to $C_m$ through $B = C_m \left( \frac{dp}{dT}\right) _{T_m}$, where $T_m$ is the mean membrane temperature, approximated as the average of the bulk feed and permeate temperatures. Under the assumption of dilute solutions, concentration effects can be neglected. Using the Clausius–Clapeyron relation^31^, the pressure–temperature derivative is expressed as

6 $$\begin{aligned} \left( \frac{dp}{dT}\right) _{T_m} = \left[ \left( \frac{\Delta H_v}{R T^2}\right) p_0(T)\right] _{T_m}, \end{aligned}$$

where $\Delta H_v$ is the latent heat of vaporization, *R* the universal gas constant, and $p_0(T)$ the vapor pressure of water, computed using the Antoine equation: $p_0(T) = 6.11 \times 10^{\tfrac{7.5 T}{237.7+T}} \, [\textrm{mbar}]$, with *T* in Celsius degrees.

The value of $C_m$ depends on the dominant transport mechanism in the membrane pores. Three possible mechanisms are considered: Knudsen diffusion, viscous (Poiseuille) flow, and molecular diffusion. Knudsen diffusion dominates when the pore size is smaller than the molecular mean free path, while molecular diffusion arises when molecule–molecule collisions prevail. In the case of DCMD under atmospheric pressure, viscous flow is negligible and usually disregarded^32^. The determining parameter is the Knudsen number *Kn*, defined as the ratio of mean free path $\lambda$ to pore diameter $d_P$:

7 $$\begin{aligned} Kn = \frac{\lambda }{d_P}, \qquad \lambda = \frac{k_B T_m}{\sqrt{2 \pi P_m \sigma _v^2}}, \end{aligned}$$

where $k_B$ is the Boltzmann constant, $P_m$ the mean pressure inside the pores, and $\sigma _v$ the collision diameter of water vapor molecules ($\sigma _v \approx 2.641 \, {{\AA }}$^33^).

If $Kn > 1$, Knudsen diffusion prevails and the permeability coefficient is

8 $$\begin{aligned} C_m = C_{kn} = \frac{2M_w}{3RT} \frac{\varepsilon r}{\tau _m \delta } \left( \frac{8RT}{\pi M_w}\right) ^{1/2}, \end{aligned}$$

where $\varepsilon$ is the porosity, *r* the pore radius, $\delta$ the membrane thickness, $M_w$ the molecular weight of water vapor, and $\tau _m$ the tortuosity, estimated as $\tau _m = (2-\varepsilon )^{2}/\varepsilon$.

If $Kn < 0.01$, molecular diffusion dominates^34^, and

9 $$\begin{aligned} C_m = C_D = \varepsilon \frac{PD}{p_{\textrm{air}}} \frac{M_w}{RT} \frac{1}{\tau _m \delta }, \end{aligned}$$

where *P* is the total pore pressure, $p_{\textrm{air}}$ the partial air pressure, and *D* the diffusion coefficient. For air–water vapor systems, *PD* can be estimated as

10 $$\begin{aligned} PD = 1.895 \times 10^{-5} T^{2.072} \, [\text {Pa}\cdot \text {m}^2/\text {s}]. \end{aligned}$$

For intermediate values $0.01< Kn < 1$, transport occurs in the transition regime and Knudsen and molecular diffusion act in series. An electrical resistance analogy is applied, where $R_{kn} = \frac{1}{C_{kn}}$, $R_D = \frac{1}{C_D}$, and

11 $$\begin{aligned} R_T = R_{kn} + R_D, \qquad C_T = \frac{1}{R_T}. \end{aligned}$$

In the case study considered here, $Kn = 0.60$, corresponding to transition conditions. The effective mass transfer coefficient is therefore

12 $$\begin{aligned} B = C_T \left( \frac{dp}{dT}\right) _{T_m} = \frac{1}{R_T} \left[ \left( \frac{\Delta H_v}{RT^2}\right) p_0(T)\right] _{T_m}. \end{aligned}$$

In the simulations, Eq. 12 is used to compute the permeate flux. The required inputs are the mass transfer coefficient *B* and the bulk permeate temperature $T_{b,p}$. Since the membrane surface temperatures $T_{m,f}$ and $T_{m,p}$ are not known a priori, the mean membrane temperature $T_m$ is approximated as the average of the feed and permeate bulk temperatures.

## Computational methods

### Fluid dynamics model

The governing equations of the modeled DCMD process are based on balance of mass, momentum, and energy for compressible fluids.

Mass conservation is expressed by the continuity equation:

13 $$\begin{aligned} \frac{\partial \rho }{\partial t} + \nabla \cdot (\rho \textbf{v}) = 0, \end{aligned}$$

where $\rho$ is the fluid density, *t* is time, and $\textbf{v}$ is the velocity vector. The first term denotes the local rate of density change, while the second accounts for advective transport.

Momentum balance follows Newton’s second law:

14 $$\begin{aligned} \frac{\partial (\rho \textbf{v})}{\partial t} + \nabla \cdot (\rho \textbf{v}\textbf{v}) + \nabla p + \nabla \cdot \boldsymbol{\tau _f} = \rho\textbf{f}, \end{aligned}$$

where *p* is pressure, $\boldsymbol{\tau _f}$ the viscous stress tensor, and $\textbf{f}$ a body force per unit mass. The terms respectively describe the time rate of momentum, advection, pressure forces, viscous forces, and external body forces. For Newtonian fluids,

15 $$\begin{aligned} \boldsymbol{\tau _f} = -\mu \left( \nabla \textbf{v} + \nabla \textbf{v}^T \right) + \left( \tfrac{2}{3}\mu - k\right) (\nabla \cdot \textbf{v}) \textbf{I}, \end{aligned}$$

with $\mu$ the dynamic viscosity, *k* the bulk viscosity, and $\textbf{I}$ the identity tensor.

Energy conservation is based on the first law of thermodynamics:

16 $$\begin{aligned} \frac{\partial (\rho e)}{\partial t} = - \nabla \cdot (\rho e \textbf{v}) - \nabla \cdot \textbf{q} - \nabla \cdot (p \textbf{v}) + \rho \textbf{f} \cdot \textbf{v} - \nabla \cdot (\boldsymbol{\tau _f} \cdot \textbf{v}) + \varphi _s, \end{aligned}$$

where $e = u + \tfrac{1}{2}|\textbf{v}|^2$ is the total energy per unit mass (sum of internal energy *u* and kinetic energy), $\textbf{q}$ is the conductive heat flux, and $\varphi _s$ represents internal heat generation. The terms correspond to energy accumulation, advection, heat conduction, pressure work, work of body forces, viscous dissipation, and internal sources, respectively.

To incorporate phase change at the membrane, the equations are modified by adding volumetric source terms for vapor mass flux, denoted as $J^\mathrm{v}$ :

17 $$\begin{aligned} \frac{{\partial }\rho }{{\partial }t}+{\nabla }\cdot \left( \rho \textbf{v} \right) =J^\mathrm{v}, \end{aligned}$$

18 $$\begin{aligned} \frac{{\partial }\rho _1}{{\partial }t}+{\nabla }\cdot \left( \rho _1\textbf{v}\right) =0, \end{aligned}$$

19 $$\begin{aligned} \frac{{\partial }( \rho \textbf{v} )}{{\partial }t}=-{\nabla }\cdot \left( \rho \textbf{vv} \right) -{\nabla }p+\mu {\nabla }^2\textbf{v}+J^\mathrm{v}\textbf{v}, \end{aligned}$$

20 $$\begin{aligned} \frac{{\partial }}{{\partial }t}\left( \rho e \right) =-{\nabla }\cdot \left( \rho e \textbf{v}\right) -{\nabla }\cdot \textbf{q}-{\nabla }\cdot p\textbf{v}+\rho \textbf{f}\cdot \textbf{v}-{\nabla }\cdot \left( \boldsymbol{\tau _f} \cdot \textbf{v}\right) +J^\mathrm{v} \Delta H_v. \end{aligned}$$

Here, $\rho _1=\Phi \rho$ is the salt density, $\Phi$ the mass fraction of solute, and $\Delta H_v$ the latent heat of vaporization. The *J*^v^ term in Eq. 17 represents the removal of water vapor through the membrane; the $J^\mathrm{v}\textbf{v}$ term in Eq. 19 removes the momentum of evaporated water to prevent spurious fluid acceleration in the feed channel; and the $J^\mathrm{v}\Delta H_v$ term in Eq. 20 accounts for evaporative cooling on the feed side.

The thermophysical properties of the aqueous salt solution are modeled as linear functions of the solute mass fraction $\Phi$. Property values at $\Phi =0$ and $\Phi =1$ (kg/kg) are used to interpolate the intermediate values at each point in space and time. For instance, dynamic viscosity $\mu$ is calculated as $\mu = \mu _0 (1-\Phi ) + \mu _1 \Phi$, where $\mu _0$ and $\mu _1$ are the viscosities at $\Phi =0$ and $\Phi =1$, respectively. Thus, $\mu =\mu _0$ for $\Phi =0$ and $\mu =\mu _1$ for $\Phi =1$. For vapor pressure, the dependence on salt concentration is neglected in this study.

### Heat transfer model

The heat transfer model takes inspiration from the assumptions of Albeirutty et al.^8^, which is used for validating the model developed in this work. In that study, the DCMD device consisted of two narrow channels separated by a polycarbonate sheet. A spacer was placed in the feed channel, where a thermochromic liquid crystal (TLC) sheet adhered to the polycarbonate surface enabled measurement of feed-side surface temperature.

Therefore, heat transfer occurs through three mechanisms: convection from the hot feed solution to the TLC surface, $q_{f,\textrm{conv}}$; conduction through the TLC and polycarbonate sheets, $q_{\textrm{PC},\textrm{cond}}$; convection from the polycarbonate sheet to the cold permeate, $q_{p,\textrm{conv}}$. At steady state, these contributions are equal:

21 $$\begin{aligned} q = q_{f,\textrm{conv}} = q_{\textrm{PC},\textrm{cond}} = q_{p,\textrm{conv}} . \end{aligned}$$

Applying Newton’s law of cooling gives

22 $$\begin{aligned} q_{f,\textrm{conv}} = h_f (T_{b,f}-T_{m,f}), \hspace{0.5cm} q_{p,\textrm{conv}} = h_p (T_{m,p}-T_{b,p}), \end{aligned}$$

where $h_f$ and $h_p$ are the convective heat transfer coefficients in the feed and permeate channels. Fourier’s law for conduction through the TLC and polycarbonate sheets yields

23 $$\begin{aligned} q_{\textrm{PC},\textrm{cond}} = \frac{T_{m,f}-T_{m,p}}{\tfrac{l_{\textrm{TLC}}}{k_{\textrm{TLC}}}+\tfrac{l_{\textrm{PC}}}{k_{\textrm{PC}}}}, \end{aligned}$$

where $l_{\textrm{TLC}}$, $k_{\textrm{TLC}}$ and $l_{\textrm{PC}}$, $k_{\textrm{PC}}$ are the thickness and thermal conductivity of the TLC and polycarbonate sheets, respectively. Combining the above relations gives:

24 $$\begin{aligned} q = \frac{T_{b,f}-T_{m,f}}{1/h_f} = \frac{T_{m,f}-T_{b,p}}{\tfrac{l_{\textrm{TLC}}}{k_{\textrm{TLC}}}+\tfrac{l_{\textrm{PC}}}{k_{\textrm{PC}}}+1/h_p}, \end{aligned}$$

from which the feed-side heat transfer coefficient can be expressed as

25 $$\begin{aligned} h_f = \frac{T_{m,f}-T_{b,p}}{(T_{b,f}-T_{m,f})\left( \tfrac{l_{\textrm{TLC}}}{k_{\textrm{TLC}}}+\tfrac{l_{\textrm{PC}}}{k_{\textrm{PC}}}+1/h_p\right) }. \end{aligned}$$

The permeate-side coefficient $h_p$ is taken from the same reference work, which adopts an experimentally-validated correlation^8^:

26 $$\begin{aligned} h_p = \frac{7.54 \, k_p}{D_p}, \end{aligned}$$

where 7.54 is the Nusselt number for laminar flow between parallel plates at constant wall temperature, $k_p$ is the thermal conductivity of the permeate at the film temperature, and $D_p$ the hydraulic diameter of the permeate channel. At each time step, the heat flux from the permeate into the membrane is assumed equal to conduction into the feed channel:

27 $$\begin{aligned} q = h_p (T_{b,p}-T_{m,f}) = \frac{k}{0.5\Delta x}(T_{m,f}-T_{1}), \end{aligned}$$

where $\Delta x$ is the mesh size and $T_1$ the fluid temperature in the first feed-side cell. Solving for the membrane surface temperature gives:

28 $$\begin{aligned} T_{m,f} = \frac{h_p T_{b,p} + \tfrac{kT_1}{0.5\Delta x}}{h_p + \tfrac{k}{0.5\Delta x}} . \end{aligned}$$

The hydraulic diameters of the feed and permeate channels are both taken as $D_f = D_p = 2H$, where *H* is the half-channel height. The respective Reynolds numbers are then

29 $$\begin{aligned} \textrm{Re}_f = \frac{v_f D_f}{\nu _f}, \hspace{1cm} \textrm{Re}_p = \frac{v_p D_p}{\nu _p}, \end{aligned}$$

where $v_f$ and $v_p$ are the average velocities and $\nu _f$, $\nu _p$ the kinematic viscosity of feed and permeate, respectively, evaluated at average film temperature. The feed velocity $v_f$ is defined with respect to the unobstructed channel cross-section (neglecting spacer volume) to allow comparison with a spacer-free case. The volume averaged feed-side heat transfer coefficient $\overline{h}_f$ is expressed via the Nusselt number:

30 $$\begin{aligned} \textrm{Nu}_f = \frac{\overline{h}_f D_{h,f}}{k_f}, \end{aligned}$$

where $D_{h,f}$ is the hydraulic diameter and $k_f$ the thermal conductivity of the hot fluid at film temperature.

Finally, efficiency loss is quantified by the coefficient $\tau$:

31 $$\begin{aligned} \tau = 1 - \textrm{TPC} = \frac{T_{b,f}-T_{m,f}}{T_{b,f}-T_{b,p}}, \end{aligned}$$

where $\textrm{TPC}$ is the temperature polarization coefficient.

### Computational implementation

The Navier-Stokes equations cannot generally be solved analytically due to their non-linear nature^35^, and are therefore discretized in both space and time.

Here, spatial discretization is performed on a uniform Cartesian mesh, consisting of a single homogeneous block with isotropic cells. This transforms the governing PDEs into a system of ODEs in time, with spatial variations represented at discrete mesh points. Time integration is then carried out using an explicit Runge-Kutta scheme^36^, while the finite volume method is applied for spatial discretization^37^. The drNum (Dual Resolution Numerics) code^38–40^ is adopted as computational solver. DrNum is a GPU-optimized CFD software developed by enGits GmbH and numrax GmbH, designed for high-fidelity flow simulations with exceptional computational efficiency. It employs a dual-resolution numerical framework combining local fine-grid solvers with global efficiency, supporting compressible, multi-species, and unsteady flows. Widely used in aerospace applications, it enables automated, high-accuracy simulations on affordable GPU-based systems.

To approximate incompressible flow, a pseudo-compressibility approach is adopted, linking pressure and density through an artificial speed of sound *a*, set to ten times the maximum fluid velocity:

32 $$\begin{aligned} \rho = \rho _0 + \frac{(p - p_0)}{a^2}. \end{aligned}$$

This ensures small density variations while preserving pressure–density coupling. Larger *a* values reduce compressibility effects but increase computational cost by requiring smaller time steps. The approach is analogous to the Lattice-Boltzmann method^41^, where pressure and density are directly related and the flow remains effectively incompressible.

The computational domain was defined to represent a repeating section of a spacer-filled feed channel, rather than a full-length MD module. Specifically, the domain spans six spacer unit cells in the axial direction and four unit cells in the spanwise direction. In the axial direction, velocity inlet and pressure outlet boundary conditions were applied. We verified that the flow becomes fully developed within approximately one spacer cell; therefore, the first and last spacer cells were excluded from post-processing to minimize inlet/outlet boundary effects. In the spanwise direction, periodic boundary conditions were imposed to approximate an effectively infinite channel width.

Numerical results were checked for mesh-independence. In detail, a mesh-independence assessment was performed using the baseline two-layer cylindrical spacer (2LCY). The procedure consisted of systematically refining the uniform Cartesian grid by halving the cell size while keeping all operating conditions and boundary conditions unchanged, and monitoring the resulting pressure drop. Grid convergence was achieved with a cell size of 0.1 mm, for which further refinement produced negligible variations in pressure drop. This resolution corresponds to meshes on the order of 90 million cells (0.1 mm cell size) for each 3D computational domain.

## Results

### Model validation

**Figure 2:**
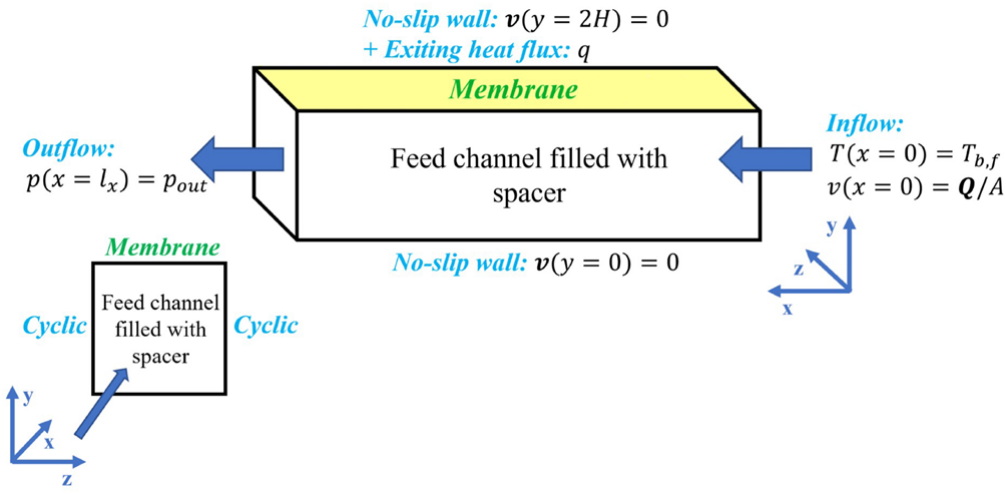
Boundary conditions applied to the computational domain representing the feed channel. The domain extends a length $l_x$ in the *x*-direction, a height 2*H* in the *y*-direction, and a unit depth in the *z*-direction.

The experimental reference study by Albeirutty et al.^8^ investigated heat transfer in a DCMD-like configuration consisting of two narrow channels separated by a polycarbonate sheet replacing the membrane, in order to isolate and quantify convective heat transfer in the spacer-filled feed channel while avoiding additional uncertainties related to membrane permeability, wetting, and phase-change mass transfer. The spacer was placed in the feed channel, where hot water flowing around the filaments promoted mixing and enhanced convective heat transfer, while cold water circulated in the permeate channel. Surface temperatures were measured using thermochromic liquid crystals attached to the polycarbonate sheet. This controlled configuration provides a clean benchmark for validating the thermo-hydrodynamic component of the model developed in this work, specifically pressure losses and heat transfer enhancement induced by the spacer, before introducing the full MD physics (evaporation and salt transport) in subsequent analyses. Thus, our numerical model was adapted to reproduce these experiments while maintaining a simplified computational setup. Only the feed channel was simulated, represented as a cuboidal domain containing the spacer, with the upper boundary modeling the membrane surface (Fig. 2).

**Figure 3:**
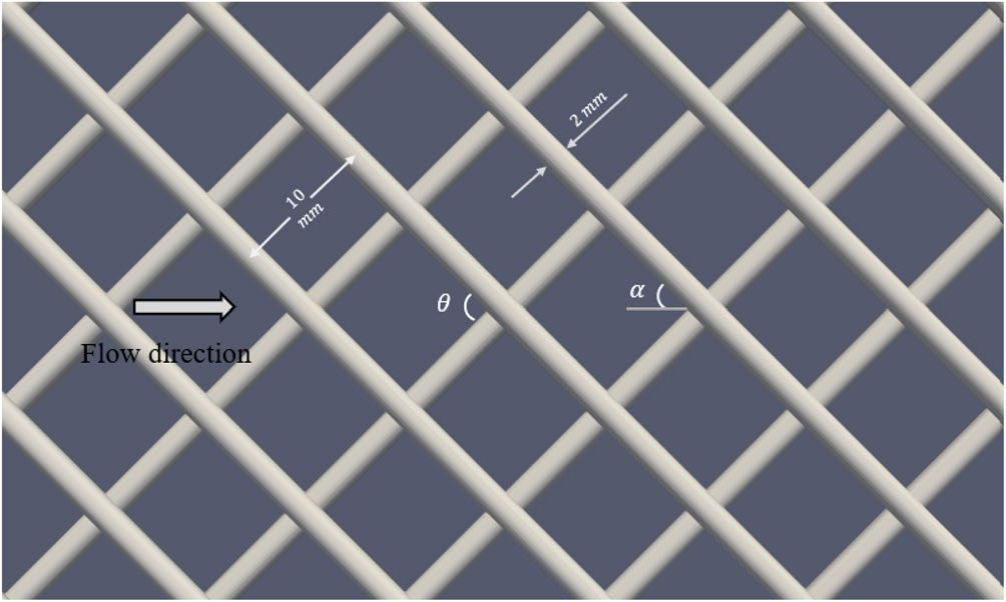
Definition of geometric parameters: attack angle $\alpha$ (shown here as $45^{\circ }$), filament crossing angle $\theta$ (here $90^{\circ }$), mesh length (10 mm), and filament diameter (2 mm).

Boundary conditions were defined as follows. Periodic (cyclic) conditions were applied to the lateral faces in the *z*-direction to represent fully developed flow in a repeating geometry. No-slip conditions were imposed at the bottom wall ($y=0$) and at the top boundary ($y=2H$), which represents the membrane surface. At this upper boundary, a Neumann condition was added to account for heat flux, combining conduction through the polycarbonate sheet and convection into the permeate channel. At the inlet ($x=0$), velocity was prescribed from the volumetric feed flow rate and inlet temperature $T_{b,f}$, while the outlet ($x=l_x$) was set to constant atmospheric pressure $p_{\textrm{out}}$.

**Figure 4:**
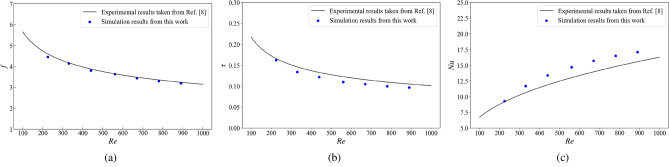
Comparison of numerical and experimental results for (**a**) Darcy friction factor, (**b**) efficiency loss coefficient, and (**c**) Nusselt number, as functions of Reynolds number, for the spacer with $\alpha =45^{\circ }$. In the model, the Nusselt number was obtained by first time-averaging the feed-side membrane temperature $T_{m,f}$, then spatially averaging it over the probe region to compute the heat transfer coefficient.

The spacer geometry tested in the validation had the following basic dimensions: a filament diameter of $2\,\textrm{mm}$, a thickness of $3.8\,\textrm{mm}$, a mesh length of $10\,\textrm{mm}$, a filament crossing angle $\theta = 90^{\circ }$, and an attack angle $\alpha =45^{\circ }$ (Fig. 3). The spacers were fabricated in ABS using a 3D printing process; upper and lower filament layers overlapped by 0.2 mm to ensure structural integrity. Coherently with experiments^8^, the validation campaign was carried out over a range of feed flow rates from 1 to 4 L/min in 0.5 L/min increments. In the simulations, inlet velocity was computed consistently from these flow rates and the channel cross-section, ensuring that only the inlet velocity and spacer geometry varied across cases.

Model performance was assessed by comparing simulations with experimental correlations for three dimensionless parameters: the Darcy friction factor *f*, the Nusselt number Nu, and the efficiency loss coefficient $\tau$, all expressed as functions of Reynolds number Re (see Fig. 4). The agreement between model and experiments is excellent: the mean absolute percentage error (MAPE) was 2.42% for *f*, 7.75% for $\tau$, and 10.13% for Nu, demonstrating that the model accurately reproduces both hydraulic resistance and heat transfer performance, and supports its use for investigating alternative spacer geometries.

### Model exploration

#### Tested spacer geometries

**Figure 5:**
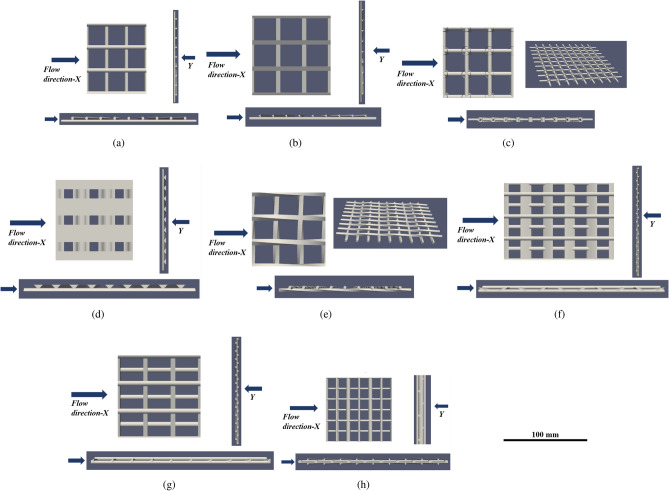
Spacer geometries investigated: (**a**) two-layer cylindrical (2LCY), (**b**) two-layer triangular (2LTR), (**c**) two-layer pillar (2LPI), (**d**) two-layer smooth cylindrical (2LSCY), (**e**) two-layer twisted (2LTW), (**f**) three-layer elliptical (3LEL), (**g**) three-layer triangular (3LTR), (**h**) five-layer cylindrical (5LCY).

Following model validation, the exploration phase focused on spacer designs that are either well-established in the literature or represent innovative alternatives with potential performance advantages. The selected geometries, illustrated in Fig. 5, cover a wide range of structural concepts, from conventional two-layer arrangements to multilayer and innovative aerodynamically inspired designs.

*Two-Layer Cylindrical Spacer (2LCY).* The 2LCY is the most widely reported configuration in the literature^2,3,7–9,11,18,28,42–54^. It consists of two orthogonal layers of cylindrical filaments arranged in a crosswise pattern. This geometry promotes fluid mixing, enhances heat and mass transfer, and has been extensively validated both experimentally and numerically. It also served as the reference case in the present validation study (“Model validation”).

*Two-Layer Triangular Spacer (2LTR).* A variant of the standard cylindrical design, the 2LTR replaces circular filaments with triangular ones^43^. The sharp edges of the triangular cross-section increase flow disruption, improve mixing, and promote a more uniform temperature field along the membrane surface, thereby reducing polarization effects. Although less common in practice, it has demonstrated promising performance and was therefore included in the present analysis.

*Two-Layer Pillar Spacer (2LPI).* The pillar spacer is a more recent concept that has attracted industrial attention^18^. It consists of regularly spaced cylindrical pillars that interrupt the flow. This arrangement reduces longitudinal pressure drop while still promoting mixing, offering potential benefits in terms of permeate flux and fouling mitigation.

*Two-Layer Smooth Cylindrical Spacer (2LSCY).* This geometry is derived from the 2LCY design but replaces full cylinders with half-cylinder cross-sections featuring smooth contours. The aim is to strike a balance between mixing enhancement and reduced hydraulic resistance. By softening the flow separation regions typical of sharp cylindrical filaments, the 2LSCY design can improve thermal performance without incurring excessive pressure losses.

*Two-Layer Twisted Spacer (2LTW).* In this configuration, cubic-section filaments are helically twisted along their axis. The twist induces secondary flows and vortical structures that enhance fluid mixing and heat transfer, while the more complex geometry may also reduce the risk of fouling. However, the improved mixing comes at the cost of increased fabrication complexity.

*Three-Layer Elliptical Spacer (3LEL).* Inspired by aerodynamic profiles, the 3LEL introduces elliptical filaments to minimize drag and limit stagnant zones. The central layer consists of elliptical filaments oriented alternately at $-20^{\circ }$ and $+20^{\circ }$, flanked by two layers of cylindrical filaments for structural support. The smoother contours reduce pressure losses while maintaining strong mixing, offering an attractive trade-off between hydraulic and thermal performance.

*Three-Layer Triangular Spacer (3LTR).* This hybrid design combines features of the triangular and elliptical geometries. The central layer comprises triangular filaments oriented perpendicular to the flow, while the outer layers consist of cylindrical filaments aligned with the flow direction. This arrangement is intended to maximize mixing in the core of the channel while keeping pressure losses manageable at the boundaries.

*Five-Layer Cylindrical Spacer (5LCY).* This complex configuration incorporates five layers arranged in three functional sections: a central layer of cylinders oriented perpendicular to the flow, and upper and lower sections each containing two orthogonal layers (parallel and perpendicular to the flow). The interpenetration of these layers enhances mixing throughout the channel. Filament diameters vary: external cylinders occupy one-quarter of the spacer thickness, while central cylinders span half. Although hydrodynamically intensive, this design offers strong mixing and polarization reduction.

Overall, while the conventional two-layer cylindrical spacer is well established in MD and is therefore used as a reference geometry in this study, only a few studies already explored two-layer triangular spacers in MD. On the other hand, no MD-specific studies addressing the pillar-type, twisted, elliptical, or multilayer (3+ layers) spacers simulated in this work under temperature-polarization-dominated MD conditions are currently available in the literature, to the best of the Authors’ knowledge. In detail, multilayer and non-cylindrical filament spacers have been studied in pressure-driven processes but remain largely unexplored in MD. Table 1 summarizes the main bulk geometric properties of the investigated spacers.

**Table 1:** Bulk geometric properties of the investigated spacer configurations.

Spacer	Layers	$D_h$ [mm]	Porosity [-]
2LCY	2	5.01	0.78
2LTR	2	5.71	0.88
2LPI	2	4.87	0.88
2LSCY	2	1.99	0.52
2LTW	2	4.43	0.72
3LEL	3	1.79	0.51
3LTR	3	2.56	0.61
5LCY	5	3.22	0.72

The effective hydraulic diameter $D_h$ is computed from the unobstructed flow area and wetted perimeter of the spacer-filled channel^55^.

#### Temperature polarization

**Figure 6:**
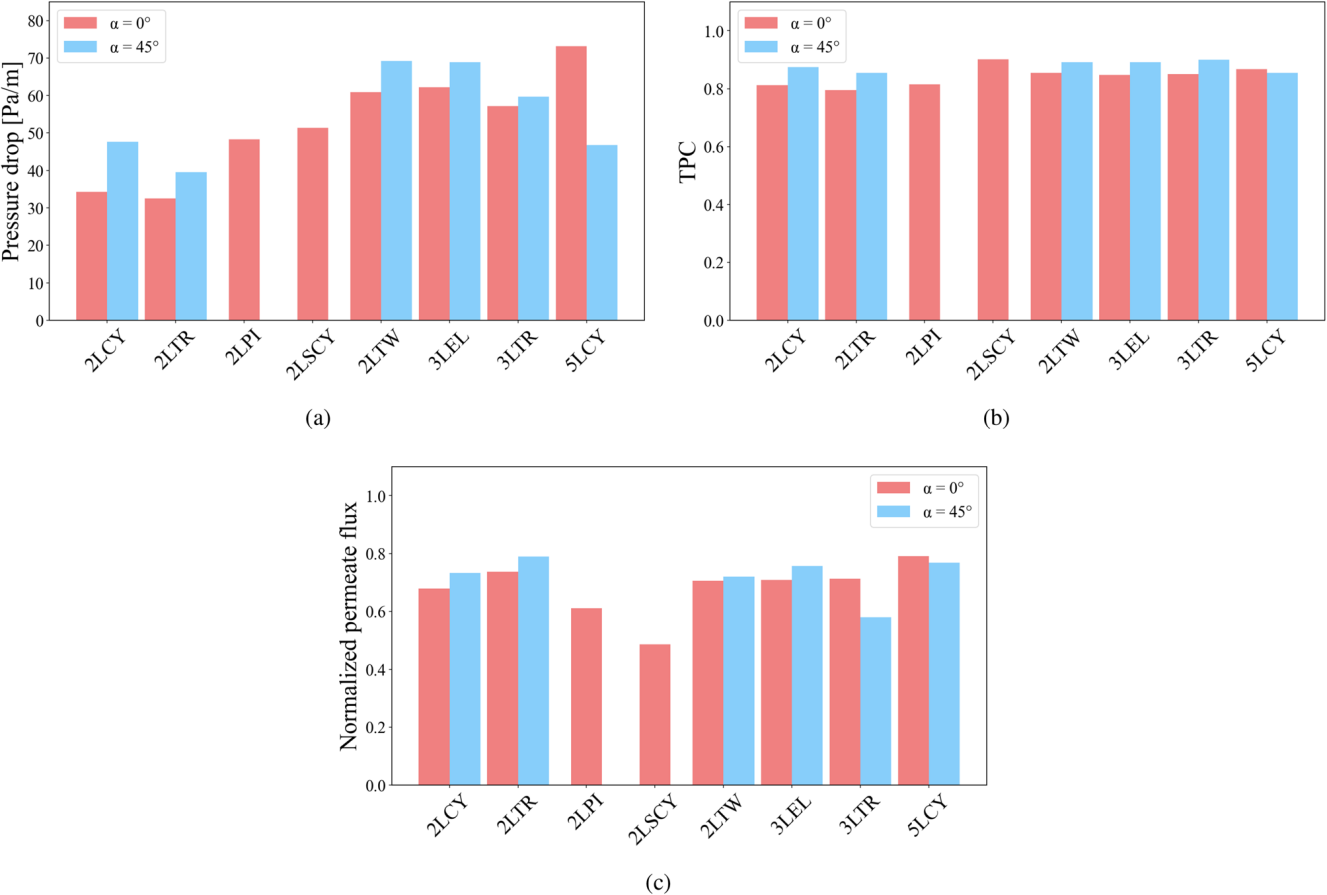
(**a**) Pressure drop, (**b**) temperature polarization coefficient (TPC), and (**c**) normalized permeate flux for spacer flow attack angles of $\alpha = 0^\circ$ (red) and $\alpha = 45^\circ$ (blue). All spacers have a mesh length of 16 mm, filament diameter of 3 mm, and thickness of 6 mm.

At this stage of the study, the analysis focused exclusively on temperature polarization, as the primary objective was to screen spacer geometries based on their thermal performance. Salt transport, and thus concentration polarization, was not yet included in the model. Model outcomes are presented in Fig. 6, where results related to flow attack angles of $\alpha = 0^\circ$ are highlighted in red and the ones related to $\alpha = 45^\circ$ in blue.

First, we analyze a flow attack angle of $\alpha = 0^\circ$. As shown in Fig. 6a, traditional spacers (2LCY, 2LTR, 2LPI) exhibit lower pressure drops, whereas the innovative spacers introduced in this work generally induce higher pressure drops. The maximum pressure drop occurs for the 5LCY, which can be attributed to the larger dimensions in terms of thickness compared to the other spacers. Conversely, Fig. 6b shows that the innovative spacers achieve higher TPC relative to traditional designs. Notably, the 2LSCY demonstrates superior mixing performance, resulting in the highest TPC. Although this is likely due to the extensive membrane surface coverage by the cylinder base, with a consequent restricted permeate flux, as illustrated in Fig. 6c.

Subsequent tests were performed with spacers oriented at a flow attack angle of $\alpha = 45^\circ$. According to prior literature^2,3,7–9,11,18,28,42–54^, this angle is expected to yield more favorable performance outcomes. The tested spacers retained the same geometric parameters as in the previous round, with the sole modification being the flow attack angle. Due to unsatisfactory permeate flux characteristics, the 2LPI and the 2LSCY were excluded from further evaluation, despite exhibiting TPC values comparable to or higher than the remaining spacers. This behavior is primarily governed by local geometric effects, such as membrane surface coverage and near-wall flow obstruction, and is therefore only weakly influenced by the flow attack angle. Considering $\alpha = 45^\circ$, Fig. 6a reveals a pressure drop trend similar to that observed at $\alpha = 0^\circ$, with the exception that the 5LCY exhibits reduced pressure drops relative to other spacers. Figure 6b shows a general increase in TPC values of $4.6\%$, maintaining the trend observed at $\alpha = 0^\circ$, with a maximum increase of $7.8\%$ for 2LCY and a slight decrease of $1.6\%$ for 5LCY. Concurrently, normalized permeate flux experiences a modest overall increase of $3.1\%$ (again with a maximum of $7.8\%$ for 2LCY), except for the 3LTR and 5LCY, which deviate from this trend with a decrease respectively of $1.9\%$ and $2.9\%$ (Fig. 6c). These results confirm that increasing the flow attack angle to $45^\circ$ leads to a significant enhancement in TPC and a moderate improvement in permeate flux. Therefore, all spacers examined in the subsequent sections are tested at a flow attack angle of $\alpha = 45^\circ$.

**Figure 7:**
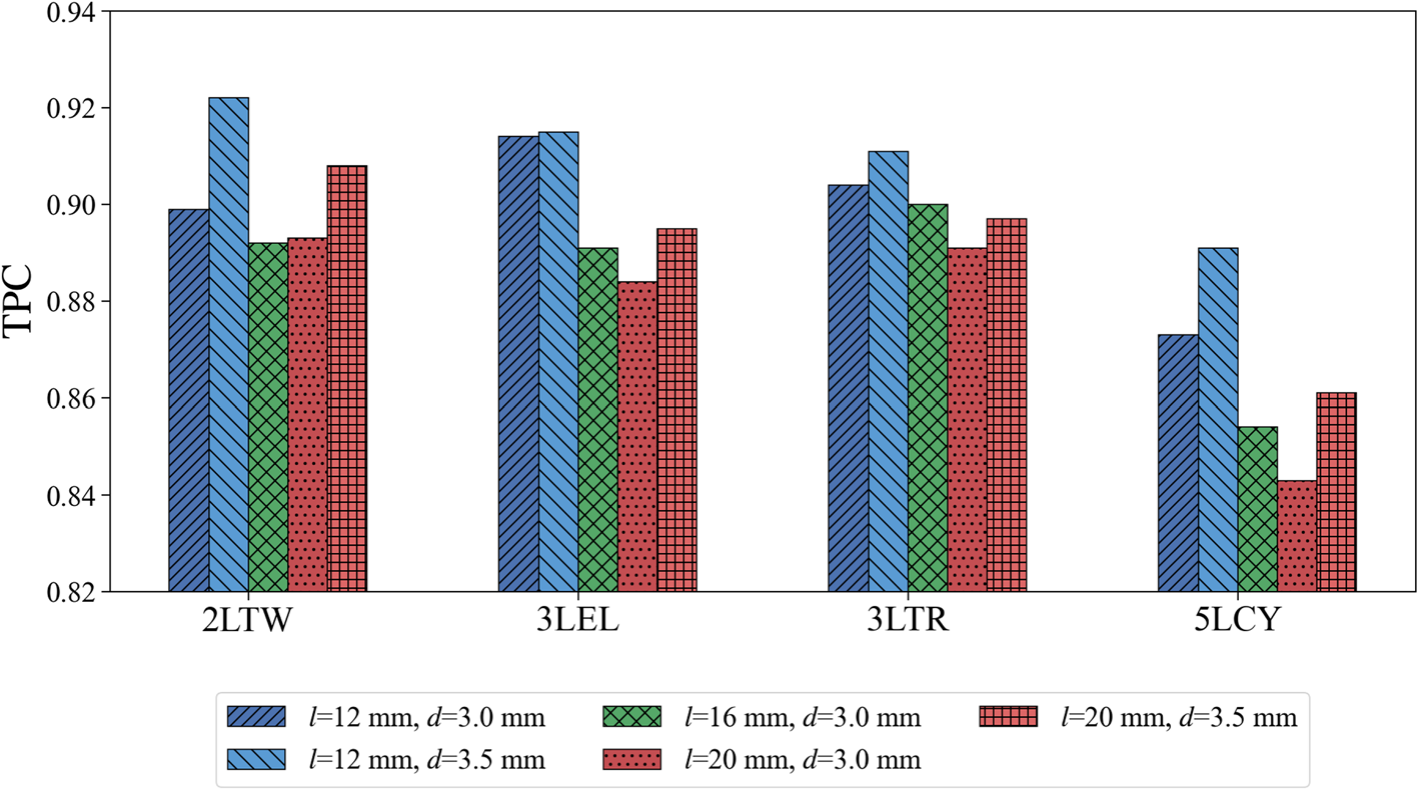
TPC results from tests with varying mesh lengths and filament diameters. Mesh lengths of 12 mm (blue), 16 mm (green) and 20 mm (red) are considered combined with diameters of 3.0 mm and 3.5 mm (see the legend).

To facilitate comparison with existing spacer studies and commonly adopted heat transfer correlations in membrane distillation, the analysis is additionally discussed in terms of dimensionless numbers. In particular, Nusselt numbers are reported alongside friction factors, following recent guidance on correlation selection for MD systems^56^. To enable a direct comparison with existing spacer studies and with commonly used heat/momentum-transfer correlations in membrane distillation, we report the Darcy friction factor *f* and the Nusselt number (Nu) for the spacer screening cases shown in Fig. 6 (all at Re=351). Following the same definitions adopted in the validation section, *f* is computed from the streamwise pressure gradient, while Nu is obtained from the hot-side convective coefficient inferred from the measured temperature polarization coefficient (TPC) and the overall thermal resistance used in the boundary condition. The resulting values are summarized in Table 2. This allows a clearer comparison across geometries and facilitates benchmarking against literature.

**Table 2:** Dimensionless comparison for the spacer screening cases in Fig. 6 (all at Re=351): Darcy friction factor *f* computed from the streamwise pressure gradient and Nusselt number (Nu) inferred from TPC using the same overall thermal resistance adopted in the boundary condition.

Spacer	$\alpha$ [$^\circ$]	$\Delta p/L$ [Pa/m]	*f* [–]	Nu [–]
2LCY	0	34.31	0.311	5.95
2LTR	0	32.53	0.295	5.34
2LPI	0	48.31	0.439	6.06
2LSCY	0	51.32	0.466	12.67
2LTW	0	60.89	0.553	8.12
3LEL	0	62.16	0.564	7.62
3LTR	0	57.17	0.519	7.86
5LCY	0	73.17	0.664	9.05
2LCY	45	47.65	0.433	10.07
2LTR	45	39.52	0.359	8.49
2LPI	45	41.39	0.376	7.56
2LTW	45	69.23	0.629	11.89
3LEL	45	68.88	0.625	11.76
3LTR	45	59.64	0.541	12.95
5LCY	45	46.72	0.424	8.42

Further simulations were conducted on the novel spacers that, with the configuration of $\alpha = 45^\circ$, demonstrated satisfying results. The selected spacers for additional testing were 2LTW, 3LEL, 3LTR, and 5LCY. In this third round, the results were primarily evaluated in terms of the temperature polarization coefficient and pressure losses; while permeate flux remains an important indicator of spacer behavior, it is largely dependent on temperature and thus follows a similar trend. More detailed permeate flux analysis, incorporating salt as a distinct species, is presented in “Concentration polarization”. To optimize spacer performance, modifications were introduced to the geometric parameters of the most promising configurations. Two key factors were varied: filament diameter and mesh length. The diameter of the spacer filaments was increased from 3 mm to 3.5 mm, while the mesh length was either reduced from 16 mm to 12 mm or increased to 20 mm. In addition to these individual changes, selected combinations of filament diameter and mesh length were also tested. All cases were simulated at a flow attack angle of $45^\circ$, yielding a total of 20 distinct spacer geometries. The complete set of tested configurations is reported in Fig. 7.

The results indicate that increasing the filament diameter from 3 mm to 3.5 mm consistently enhances the TPC across all spacer geometries, independent of mesh length. Relative to the baseline configuration (3 mm diameter, 16 mm mesh length), reducing the mesh length to 12 mm yields a clear improvement in TPC, whereas extending it to 20 mm causes a performance decline. The most favorable outcomes are therefore obtained with a mesh length of 12 mm combined with a filament diameter of 3.5 mm.

The observed trends can be explained by the hydrodynamic impact of spacer geometry on boundary layer development. Increasing the filament diameter enhances flow obstruction and induces stronger local mixing and higher shear stresses near the membrane surface, resulting in a thinner thermal boundary layer and improved heat transfer, which directly increases TPC. Similarly, reducing the mesh length increases the frequency of flow disruption along the channel, preventing full redevelopment of the thermal boundary layer between adjacent filaments. Conversely, larger mesh lengths allow thicker boundary layers to form, reducing convective heat transfer and leading to lower TPC.

To complement the TPC analysis, we also evaluated the associated hydraulic penalty for the optimized configurations. Considering the cases with filament diameter of 3.5 mm, the magnitude of the streamwise pressure gradient at mesh length equal to 12 mm is 266.89 Pa/m for 3LEL, 239.97 Pa/m for 2LTW, 143.66 Pa/m for 3LTR, and 89.45 Pa/m for 5LCY. When the mesh length is increased to 20 mm, the corresponding values decrease to 92.15, 104.75, 64.07, and 50.11 Pa/m, respectively. Thus, the reduction of mesh length from 20 mm to 12 mm, while beneficial for TPC, leads to a marked increase in hydraulic resistance, with pressure gradient increments of about 190% for 3LEL, 129% for 2LTW, 124% for 3LTR, and 79% for 5LCY. This trend is physically consistent with the thermal behavior discussed above. A shorter mesh length increases the number of flow-disturbing elements per unit channel length, thereby enhancing mixing and suppressing thermal boundary layer redevelopment, which improves TPC. However, the same mechanism also intensifies contraction-expansion losses, viscous dissipation, and form drag, thereby increasing pressure drop. Among the investigated geometries, 3LEL and 2LTW provide the strongest thermal enhancement but also the highest hydraulic penalty, whereas 5LCY shows the lowest pressure drop at the expense of weaker heat transfer enhancement. The 3LTR configuration represents an intermediate compromise between polarization reduction and hydraulic resistance.

Accordingly, despite their higher hydraulic penalty, the 2LTW with $\alpha = 45^\circ$, 3.5 mm filament diameter, and 12 mm mesh length, together with the 3LEL in both the 3.5 mm and 3 mm filament variants at 12 mm mesh length, were retained as the most promising candidates because they achieved the highest TPC values among the tested configurations. These geometries were therefore selected for the final stage of the study, in which concentration polarization effects were incorporated to evaluate their behavior under realistic salinity conditions.

#### Concentration polarization

Building on the results of the previous section, two spacer geometries were selected for further investigation related to concentration polarization: the 2LTW and the 3LEL. These designs were subjected to an extended series of simulations in which operating conditions were systematically varied to assess their performance under realistic desalination scenarios. Each geometry was tested under two thermal conditions, corresponding to bulk feed temperatures of 313 K and 333 K, with the permeate side maintained 13 K lower (300 K and 320 K, respectively). In addition, inlet velocity components along *x* of 0.01 m/s and 0.1 m/s were imposed to explore the influence of flow rate. The solute concentration at the channel inlet was fixed at 35 g/L, representing seawater conditions. The complete set of operating cases and associated Reynolds numbers is summarized in Table 3.

**Table 3:** Overview of simulation cases considering salt transport as well, detailing the Reynolds number (Re), feed and permeate bulk temperatures ($T_{b,f}$ and $T_{b,p}$), and the *x*-component of the inlet velocity (*v*) corresponding to each test condition.

Test	1	2	3	4
Re	36.8	36.5	368.9	365.5
$T_{b,f}$ [K]	313	333	313	333
$T_{b,p}$ [K]	300	320	300	320
*v* [m/s]	0.01	0.01	0.1	0.1

**Figure 8:**
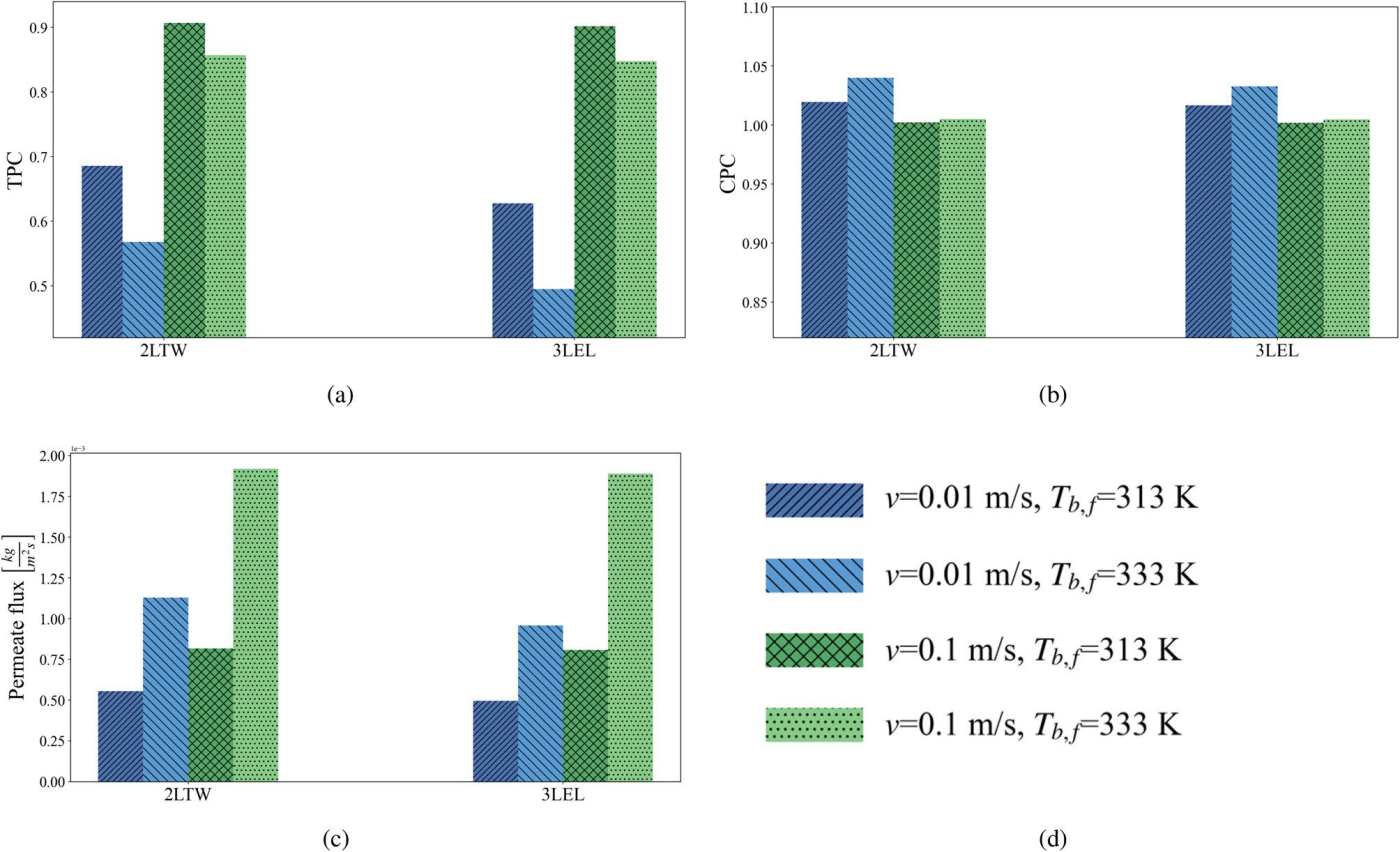
(**a**) TPC, (**b**) CPC, and (**c**) permeate flux results for 2LTW and 3LEL with a (**d**) legend for distinguishing the tests gathered in Table 3. They consider alternatively feed channel temperatures of 313 K and 333 K and inlet feed velocity *x*-components of 0.01 m/s and 0.1 m/s.

Each geometry was simulated within a 2 mm-thick domain, yielding a total of eight test cases. The performance analysis focused on three key metrics: temperature polarization coefficient (TPC), concentration polarization coefficient (CPC), and permeate flux. Unlike earlier stages, salt transport was explicitly included, enabling the model to capture concentration polarization and provide a more realistic estimate of permeate flux. Consequently, flux values are reported in absolute terms rather than normalized form.

The results for both spacer geometries are presented in Fig. 8. When evaporation and salinity effects are incorporated, a slight reduction in TPC is observed for both configurations. For instance, the best performing 3LEL (Test 3) shows a decrease of 1.2%, while the 2LTW (Test 3) records a reduction of 1.41%. Despite these small losses, the spacers remain effective in reducing polarization.

A closer examination of the trends reveals a behavior consistent with established theoretical expectations^32^. Feed temperature emerges as a decisive parameter: higher values increase the vapor pressure and thus the driving force for mass transfer, leading to higher permeate fluxes. However, this also exacerbates temperature polarization due to enhanced evaporation at the membrane interface. Similarly, feed velocity strongly influences boundary layer development. At higher velocities, the thermal and solute boundary layers are thinned, aligning surface conditions more closely with bulk values. This results in reduced polarization and increased flux, confirming the dual benefit of higher hydrodynamic shear.

**Figure 9:**
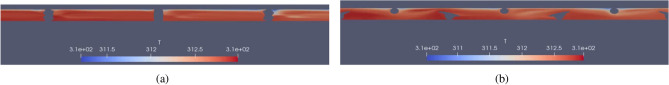
Temperature field for the spacers (**a**) 2LTW and (**b**) 3LEL, both in the condition of 0.1 m/s inlet velocity and 313 K feed channel temperature.

Comparing the two geometries, the 2LTW consistently delivers superior performance in terms of TPC and permeate flux, whereas the 3LEL achieves slightly better CPC values. Nevertheless, the temperature field contours reveal that the 3LEL (Fig. 9b) leads to a less uniform thermal distribution, with lower temperatures adjacent to the membrane surface. This indicates reduced heat transfer efficiency compared to the 2LTW (Fig. 9a), where the flow promotes better mixing and higher local temperatures near the membrane.

**Figure 10:**
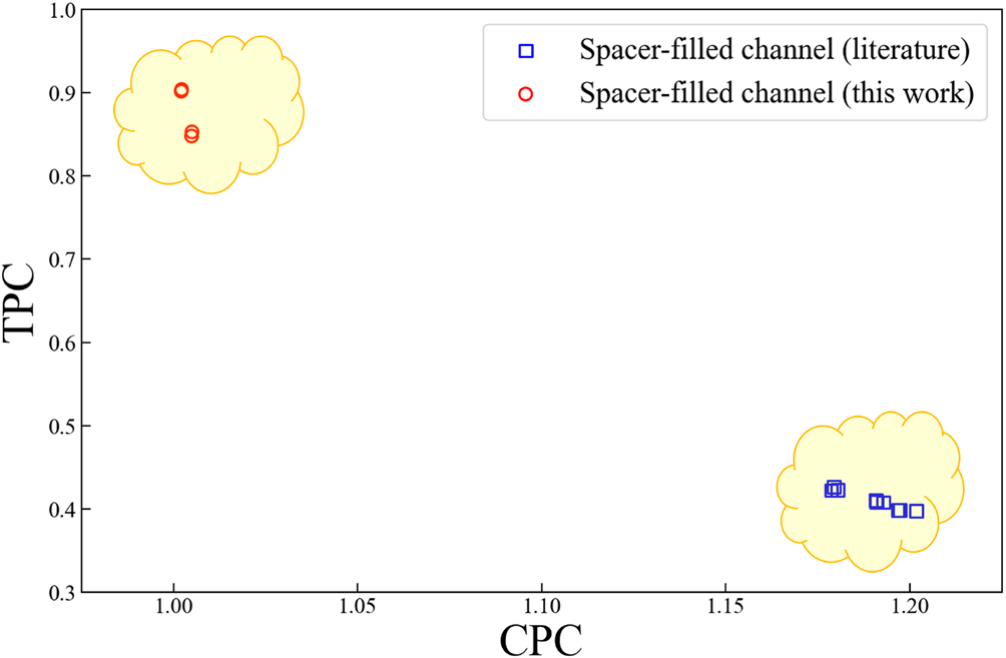
Comparison of TPC and CPC results for high performance spacers identified in this study with literature data at comparable Reynolds numbers (Re $\sim 350$). Only Tests 3 and 4 for both 2LTW and 3LEL are considered from this paper, while the literature data is taken from Ref.^57^, which analyzes semicircular, squared and triangular spacers.

To contextualize these findings, Fig. 10 compares the present results with data from the literature at comparable Reynolds numbers (Re $\sim 350$)^57^. The corresponding numerical values are reported in Table 4 to improve the interpretability of the comparison. In addition to spacer-filled channel studies, we also include representative empty-channel results from Martínez-Díez and Vázquez-González^58^, who reported both temperature and concentration polarization coefficients under laminar DCMD conditions. Empty-channel data are introduced as limiting hydrodynamic reference cases rather than as direct geometric comparisons. The scatter plot demonstrates that the novel spacer designs identified here occupy a more favorable region in the TPC–CPC space, outperforming previously reported spacer geometries and exhibiting substantially reduced polarization effects, improving TPC by up to 5.4% over the best literature geometries.

**Table 4:** Numerical values of TPC and CPC for spacer geometries investigated in this work, literature spacer data from Kuang et al.^57^, and spacer-free reference cases from Martínez-Díez et al.^58^.

Spacer geometry	Source	Re	TPC [-]	CPC [-]
2LTW (Test 3)	This work	368.9	0.907	1.002
2LTW (Test 4)	This work	365.5	0.857	1.005
3LEL (Test 3)	This work	368.9	0.902	1.002
3LEL (Test 4)	This work	365.5	0.848	1.005
Semicircular ($h=0.5$ mm)	Kuang et al.^57^	358	0.398	1.202
Semicircular ($h=0.75$ mm)	Kuang et al.^57^	358	0.408	1.193
Semicircular ($h=1$ mm)	Kuang et al.^57^	358	0.422	1.179
Squared ($h=0.5$ mm)	Kuang et al.^57^	358	0.399	1.197
Squared ($h=0.75$ mm)	Kuang et al.^57^	358	0.410	1.191
Squared ($h=1$ mm)	Kuang et al.^57^	358	0.426	1.179
Triangular ($h=0.5$ mm)	Kuang et al.^57^	358	0.398	1.197
Triangular ($h=0.75$ mm)	Kuang et al.^57^	358	0.409	1.191
Triangular ($h=1$ mm)	Kuang et al.^57^	358	0.423	1.180
Empty channel	Martìnez-Dìez et al.^58^	280	0.406	1.040
Empty channel	Martìnez-Dìez et al.^58^	436	0.438	1.040
Empty channel	Martìnez-Dìez et al.^58^	593	0.448	1.040

Empty-channel results are included as hydrodynamic reference limits. Reynolds numbers for Martínez-Díez cases were estimated from reported velocities and channel geometry. In Kuang et al., *h* denotes spacer characteristic length.

In summary, the 3LEL emerges as the preferred option in applications where concentration polarization is the dominant limiting factor, while the 2LTW represents the optimal choice when overall thermal performance and flux maximization are prioritized. Together, these results highlight the potential of tailored spacer geometries to balance competing polarization phenomena and enhance the performance of DCMD systems.

## Conclusions

This study developed a CFD model for simulating direct contact membrane distillation, with the objective of designing and evaluating innovative spacer geometries that minimize polarization phenomena and enhance process performance.

The governing balance equations for momentum, energy, and mass were implemented with spatial and temporal discretization and validated against experimental data from the literature. The model reproduced pressure drop and temperature polarization trends with mean absolute percentage errors below 3% for hydrodynamic parameters and below 8% for thermal performance, confirming its predictive reliability.

Using this validated framework, a broad set of spacer geometries – both conventional and novel – was investigated. Initial screening based on thermal performance alone revealed that optimized geometries achieved improvements in the temperature polarization coefficient (TPC) of up to 5.4% compared to the best performing literature configurations. Subsequent optimization incorporated solute transport and vapor permeation, enabling simultaneous evaluation of TPC, concentration polarization coefficient (CPC), and permeate flux under varying thermal and hydrodynamic conditions.

Quantitatively, the 2LTW demonstrated the most balanced performance, achieving the highest permeate flux and TPC across all tested scenarios. The 3LEL, while slightly less effective in terms of TPC, reduced CPC by up to 2% relative to the twisted geometry, making it particularly advantageous in salinity-sensitive applications. Both designs consistently outperformed conventional two-layer cylindrical spacers, with potential flux enhancements of 5–10% depending on the operating regime.

It is worth mentioning that although the multilayer and twisted spacers exhibit excellent numerical performance, their fabrication may require advanced 3D printing techniques, which could be costly. However, advances in additive manufacturing are expected to reduce these limitations in the future. The present study focuses on identifying design principles and performance trends, leaving detailed techno-economic assessment for future work.

Overall, the results underscore the central role of spacer geometry in DCMD module efficiency. Through targeted geometric optimization, polarization effects can be mitigated and permeate flux significantly increased. Mitigation of polarization through optimized spacer geometries is also expected to reduce fouling onset by limiting local solute accumulation and supersaturation at the membrane surface, which is beneficial for long-term MD operation. Future research should extend the present model to the full MD module, including permeate-side transport, to enable system-level performance predictions and support the design of next-generation spacer architectures for sustainable desalination.

## Data Availability

The complete dataset of model results is available upon request. Requests should be sent to the Corresponding Author (Matteo Fasano, matteo.fasano@polito.it).
